# Myeloid malignancies: mutations, models and management

**DOI:** 10.1186/1471-2407-12-304

**Published:** 2012-07-23

**Authors:** Anne Murati, Mandy Brecqueville, Raynier Devillier, Marie-Joelle Mozziconacci, Véronique Gelsi-Boyer, Daniel Birnbaum

**Affiliations:** 1 Centre de Recherche en Cancérologie de Marseille, laboratoire d’Oncologie Moléculaire; UMR1068 Inserm, Institut Paoli-Calmettes, 27 Bd. Leï Roure, BP 30059, Marseille, 13273, France

## Abstract

Myeloid malignant diseases comprise chronic (including myelodysplastic syndromes, myeloproliferative neoplasms and chronic myelomonocytic leukemia) and acute (acute myeloid leukemia) stages. They are clonal diseases arising in hematopoietic stem or progenitor cells. Mutations responsible for these diseases occur in several genes whose encoded proteins belong principally to five classes: signaling pathways proteins (e.g. CBL, FLT3, JAK2, RAS), transcription factors (e.g. CEBPA, ETV6, RUNX1), epigenetic regulators (e.g. ASXL1, DNMT3A, EZH2, IDH1, IDH2, SUZ12, TET2, UTX), tumor suppressors (e.g. TP53), and components of the spliceosome (e.g. SF3B1, SRSF2). Large-scale sequencing efforts will soon lead to the establishment of a comprehensive repertoire of these mutations, allowing for a better definition and classification of myeloid malignancies, the identification of new prognostic markers and therapeutic targets, and the development of novel therapies. Given the importance of epigenetic deregulation in myeloid diseases, the use of drugs targeting epigenetic regulators appears as a most promising therapeutic approach.

## Introduction

Myeloid malignancies are clonal diseases of hematopoietic stem or progenitor cells. They result from genetic and epigenetic alterations that perturb key processes such as self-renewal, proliferation and differentiation. They comprise chronic stages such as myeloproliferative neoplasms (MPN), myelodysplastic syndromes (MDS) and chronic myelomonocytic leukemia (CMML) and acute stages, i.e acute myeloid leukemia (AML). AML can occur *de novo* (~80% of the cases) or follow a chronic stage (secondary AML). According to the karyotype, AMLs can be subdivided into AML with favorable, intermediate or unfavorable cytogenetic risk [1]. MPNs comprise a variety of disorders such as chronic myeloid leukemia (CML) and non-CML MPNs such as polycythemia vera (PV), essential thrombocythemia (ET) and primary myelofibrosis (PMF).

Molecular biology has always been important in hematology, especially myeloid malignant diseases. Currently however, except in some specific examples such as the *BCR-ABL1* fusion in CML, and *NPM1* or *FLT3* mutations in *de novo* AML, molecular data are not associated with optimal clinical and therapeutic exploitation in the clinic. This may change with the flurry of new data that are being generated. It all started with the discovery of the JAK2V617F mutation in MPNs [2-5]. Like the characterization of the BCR-ABL1 fusion kinase, which has led to the development of an efficient targeted therapy [6], this breakthrough showed how much progress can be made by the identification of a single molecular event regarding disease definition, understanding and classification, prognosis assessment, clinical monitoring and treatment. Since then, many new mutated genes have been identified. They affect various cell processes such as signaling, regulation of gene transcription and epigenetics, mRNA splicing and others. The aim of this review is not to describe these results in detail; this has been done in several excellent recently-published reviews [7-16]. Without putting emphasis on a particular gene, disease or cell process, it is more to discuss how the new data may improve our global vision of leukemogenesis and may be used for progress in at least three directions.

## Review

### Understanding molecular leukemogenesis

#### Identification of new mutations

The genetic events involved in leukemogenesis have been deciphered by using two approaches. First, genomic alterations have been identified by using karyotype analysis and DNA hybridization onto oligonucleotide arrays (SNP-arrays, array-CGH); several types of genomic profiles have been found: lack of detectable changes, uniparental disomies (UPD), losses of chromosomes or large chromosomal regions, trisomies, losses or gains of small regions or genes. Second, small gene mutations have been detected by classical Sanger sequencing [17-22] or, more recently, by the use of new technologies such as next generation sequencing (NGS) [23-31].

**Figure 1:**
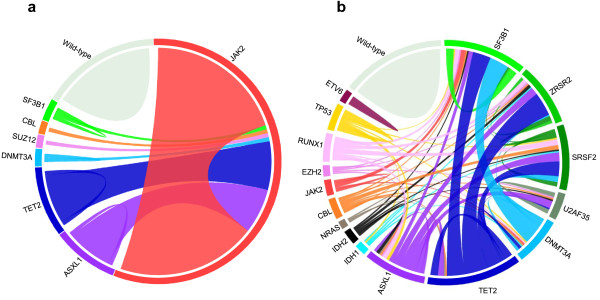
**Circos diagrams depict the relative frequency and associations of the major mutations in MPNs (a) and MDSs (b), respectively based on data from our work**[37]**on127 classic MPNs and from Damm’s study**[38]**on 221 MDSs.** Wild-type means that no disease allele has been detected in the genes listed.

These studies, together with previous ones that had identified *JAK2, NPM1, MPL, RAS* and *RUNX1* mutations, among others, led to the discovery of several major players in leukemogenesis: *ASXL1*[21], *BCORL1*[25], *CBL*[19], *DNMT3A*[24,32], *EZH2*[20,22], *IDH1/IDH2*[26], *TET2*[18] and *UTX*[33]. The mutational frequencies of these genes range from a few percent to more than 50%, or even virtually 100%, depending on the gene, the disease and the series studied. Thus, almost all cases of PV have a mutation of *JAK2*[34,35]. Not counting the latter, mutations in *ASXL1* and *TET2* are frequently observed throughout the whole myeloid spectrum (Figure 1), reaching 40-50% in CMML [33,36]. Mutations in *DNMT3A* and *IDH1/2* are rare in the chronic stages but reach 15-20% in AML and exhibit a strong association with monocytic features [30]. Genes encoding components of the splicing machinery that is involved in the splicing of introns during pre-mRNA maturation (mainly *SF3B1, SRSF2, U2AF35/U2AF1*, and *ZRSR2*) have been found frequently mutated in MDSs and CMML, and more rarely in MPNs and AML (Figure 1) [31,37-42]. Mutations in splicing factors are found in more than 60% of MDS with ring sideroblasts and in more than 50% of CMML [31].

Mutations in leukemogenic genes have been described in detail in recent reviews [7,9,10,12-16,43]; and will not be reviewed here. We will rather delve on the questions aroused by these recent data.

#### Have we already identified the entire repertoire of mutated genes?

We may have identified (most of) the major culprits [14]. First, there are hundreds of background mutations (i.e. that do not provide selective advantage) but only a limited number of driver mutations (i.e. that cause the disease) in each malignant disease. Second, many of the newly discovered mutated genes may affect the same pathways or networks as the major mutated genes. For example, deletions and mutations of *NF1*, which have been recently identified [17,44,45], or *PTPN11*[46] are thought to have the same effect as a *RAS* mutation; a mutation of the *SHKBP1* gene [47] or a duplication of the *SH3KBP1* gene [48], which both encode cytoplasmic regulators of the CBL pathway, may have the same effect as a *CBL* mutation [49,50]. Because EZH2, EED and SUZ12 proteins all belong to the same polycomb complex 2 (PRC2) the rare deletions or mutations of the *EED*[23,51] and *SUZ12* genes [17,51] could have the same effect as *EZH2* mutations. Third, several genes (e.g. *ETV6*[52] or *RUNX1*) can be structurally altered by mechanisms other than mutation, such as deletions and breakages. Fourth, some important regulatory genes could be affected not by structural alteration but through other mechanisms such as abnormal DNA methylation (e.g. *CDKN2A/B*[53], *TRIM33*[54], *CTNNA1*[55], *SOCS1*[56,57]), histone modifications, mRNA splicing, microRNA or long non-coding RNA (lncRNA) modulation, or product degradation. Fifth, when all known mutated genes are analyzed in a series of cases, the percentage of samples with at least one mutated candidate driver gene varies from 50% [58] to over 90% (in CMML; [33]; Gelsi-Boyer et al., submitted). Moreover, most samples studied by NGS were shown to harbor gene mutations [23,26]. Thus, we are soon approaching the days where all cases can be defined by combination of several alterations. The practical definition of leukemogenesis will then be based on a specific and limited repertoire of alterations, including translocations, mutations and copy number changes, affecting a defined set of driver genes.

However, some issues still need be addressed. First, many genes may be mutated or deleted with a very low frequency (i.e. under 1%); their involvement and recurrence may be hard to demonstrate. Second, because NGS studies of several malignancies have shown that hundreds of genes can be mutated in a single tumor, background mutations should be discarded and driver genes validated. Third, we still miss information in some diseases such as essential thrombocythemia (ET), in which *JAK2* mutations are found in only half the cases, and *TET2* mutations in less than 10%. We also lack knowledge about the targeted genes of some frequent genomic alterations such as the 20q11-q13 deletion (*ASXL1* and *DNMT3B*, more centromeric, are not involved). Fortunately, this lack of information is bound to disappear. The example of refractory anemia with ring sideroblasts (RARS) is instructive; in three-quarters of RARS, mutations have been recently found in *SF3B1*, a gene encoding a subunit of a splicing factor (U2 snRNP) and histone acetyltransferase (STAGA) complexes [27,29,31].

#### Is there some specificity in gene alterations?

Gene fusions (e.g. *BCR-ABL1, PML-RARA, FGFR1*-associated fusions…), 5q deletion and *JAK2* mutations are specific of some forms of myeloid diseases, although *JAK2* mutations occur in three distinct subtypes of MPN. *RUNX1* mutations are frequent in MDSs, CMML and AML but rare in MPNs. Among splicing factor genes, mutations in *SF3B1* are highly specific of MDS with ring sideroblasts and *SRSF2* mutations are most frequent in CMML [31]. In contrast, some mutated genes (e.g. *ASXL1, DNMT3A, EZH2, TET2*) occur in a wide range of myeloid diseases and with various frequencies. Future studies may identify mutations or combinations of mutations that drive a specific phenotype.

#### What are the functions of the mutated proteins ?

**Figure 2:**
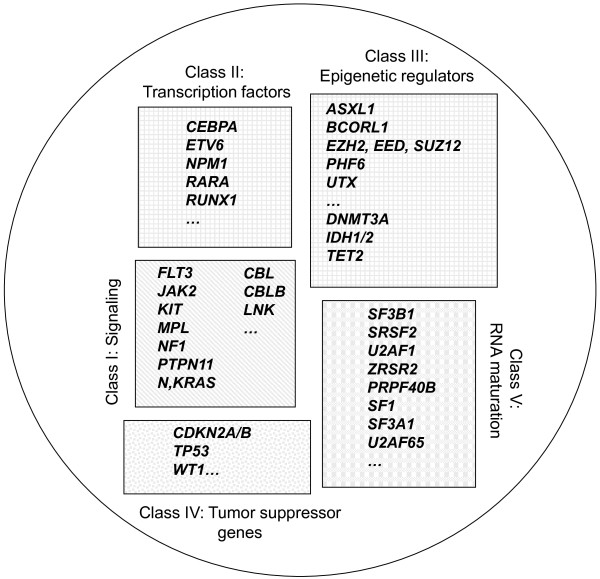
**Schematic representation of five classes of leukemogenic genes.** ERs (class III) can be subdivided into two subclasses (DNA methylation-associated and histone-associated).

Leukemogenic alterations mainly affect five classes of proteins (Figure 2): signaling pathway components, such as ABL, CBL, CBLB, FGFR1, FLT3, JAK2, KIT, LNK, MPL, PDGFRs, PTPN11, PTPRT [23,59] and RAS, transcription factors (TFs) such as CEBPA, ETV6 [58], GATA2 [30], IKZF1 [60], RARA and RUNX1, epigenetic regulators (ERs), such as ASXL1, BCORL1 [25], DAXX [23], DNMT3A, EZH2 [20,22], MLL, MYST3, NSD1 [30], PHF6 [61], SUZ12 [17,51], TET2 and UTX [28], tumor suppressors (TSG), such as CDKN2A, TP53, and WT1 and components of the spliceosome [27,29,31,38,39,41,42]. However, additional alterations occur in genes encoding proteins that it is too early to classified into these defined categories, such as DIS3, DDX41 [23], mitochondrial NAPDH dehydrogenase ND4 [62], or cohesin complex proteins [23,63].

In chronic stages, alterations in signaling molecules can be grouped in two major categories, a first one that is found in MPNs and affects oncogenic tyrosine kinases (ABL1, JAK2, FGFR1, PDGFRs) and the downstream JAK-STAT and/or PI3-kinase pathways, and a second one that is mutated in CMML and affect the RAS-MAP kinase pathway (RAS, PTPN11, NF1). CBL alterations occur in a wide variety of myeloid diseases [50].

**Figure 3:**
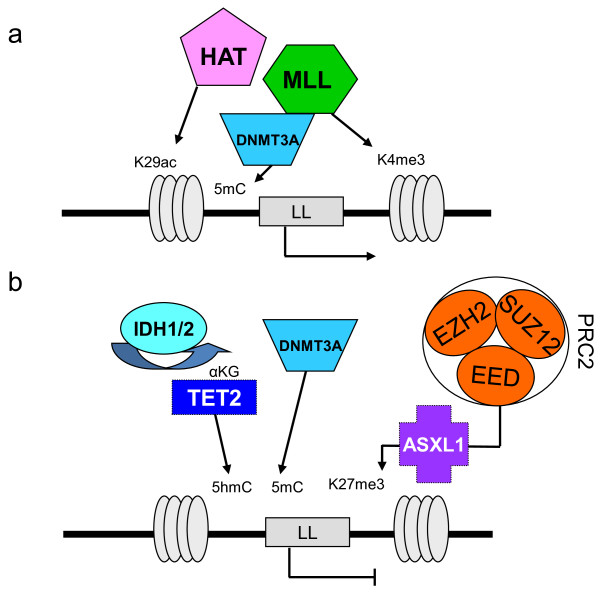
**Schematic representation of epigenetic regulation of a leukemogenic locus (LL) framed by histone H3.** (**a**) Histone acetyltransferases (HAT; e.g. MYST3) and histone methyltransferases (HMT; e.g. MLL) can activate the locus. (**b**) Reciprocally, the locus is repressed by polycomb complex PRC2 (which comprised EED, EZH2 and SUZ12 proteins). ASXL1 would direct PRC2 to the locus. Loss-of-function mutations in PRC2 components or in ASXL1 remove PRC2 repression. DNMT3A is involved in the formation of 5-methylcytosines (5mC) from cytosines and interacts with HMTs as well as with PRC2 components. TET2 mediates hydroxylation of 5mC to 5hmC. To function, TET2 requires α-ketoglutarate (αKG), which is provided by IDH1/2 proteins. Aberrant methylation patterns are caused by mutation in TET2 or in IDH1/2, which produces 2-hydroxyglutarate instead of αKG.

TFs and ERs constitute the largest classes, which involve several categories of proteins (Figure 3); because there are many ways to affect gene expression it is probable that not all of these categories are known yet. The existence of epigenetic alterations in myeloid malignancies has been known for long time [64,65]. For example, alterations of MLL, a histone methyltransferase (HMT), and MYST3, a histone acetyltransferase (HAT), have shown the importance of epigenetic deregulation in AMLs with translocation [45,64,65]. However, in chronic diseases and in AMLs with normal karyotype, the extent, causes, identities, exact roles and consequences of epigenetic alterations have long remained elusive. Molecular studies have recently shown that both DNA methylation and histone regulation are affected, and that epigenetic alterations may be due to genetic alterations, (i.e. mutations in genes encoding epigenetic regulators). The latter phenomenon has been observed in genome-wide analyses of many neoplasias [28,66,67]. However, not all epigenetic alterations may be due to an abnormal genetic background [1,53,54].

The recent reports of the interrelated functions of IDH1/2 and TET2 in DNA methylation represent a major breakthrough in our understanding of leukemogenesis [68]. It was initially hard to associate mutations of IDH1 and IDH2, two metabolic enzymes, with mutations in TET2, an unknown gene product, and as hard to suspect their role on DNA methylation. A very rapid series of elegant studies have shown i) that *IDH1/2* and *TET2* mutations are mutually exclusive in myeloid malignancies [68], ii) that mutated IDH1 and IDH2 produce 2-hydroxyglutarate instead of alpha-ketoglutarate (αKG) [69,70], iii) that *TET2* encodes an αKG–dependent methyl cytosine dioxygenase whose mutation alters the conversion of 5-methylcytosine (5-mC) to 5-hydroxymethylcytosine (5-hmC) [68,71] and iv) that both *IDH1/2* and *TET2* mutations impact on DNA methylation and are involved in the same biochemical pathway [72]. In addition, TET proteins can generate from 5-hmC 5-formylcytosine and 5-carboxylcytosine, but their roles are currently unknown [73]. The recent studies on TET proteins suggest a role in removing aberrant DNA methylation to ensure DNA methylation fidelity [74]. This has opened a new area of research since first, other factors involved in DNA demethylation may exist and second, several αKG–dependent enzymes, such as jumonji histone demethylases [75] are epigenetic regulators; therefore, some of these proteins could also be involved in malignancies. However, *IDH1/2* and *TET2* mutations, while mutually exclusive, are not equivalent because *IDH1/2* mutations are more frequent in acute than in chronic myeloid diseases, whereas it is not the case for *TET2* alterations, which are more evenly distributed between chronic and acute stages. Inactivation of TET2 increases self-renewal in hematopoietic stem cells and induces a disease resembling CMML in mouse models [76,77]. Mutated IDH1/2 enzymes may impact on self-renewal but with a different strength. The likely explanation is that IDH1/2 and TET2 have other, non-overlapping functions on the regulation of DNA methylation and histone marks. Also, an IDH-mutated product may depend on another, rate-limiting factor to exert a leukemogenic effect. DNMT3A is a *de novo* DNA methyltransferase involved in the formation of 5-mC and has complex interactions with polycomb and HMT proteins [78]. How DNMT3A mutations affect DNA methylation remains to be defined [24,30,79]; they probably do so in a different way from *TET2* or *IDH1/2* mutations since they may co-occur with either of them. A recent study showed that DNMT3A loss leads to upregulation of hematopoietic stem cell genes and downregulation of differentiation genes but is alone insufficient to induce a malignant disease in a mouse model [79].

Mutations in regulators of histone marks have become a major subject of research and the relationships between them are quickly unveiled. Central regulators of myelopoiesis and key players in leukemogenesis seem to be the polycomb regulatory complexes, especially PRC2, which, in addition to direct defects of its components (EED, EZH2, SUZ12), could be affected in its concerted action with several ERs, such as ASXL1, cohesins, DNMT3A, IDH1/2, MLL, TET2 and UTX. TET proteins could regulate pluripotency and self-renewal through interaction with PRC2 [74,80,81]. The cohesin complex is encoded by four genes (*SMC1, SMC3, RAD21* and *STAG2*), which have been found mutated [23] and deleted [63]. A major interactor of cohesin complex is CTCF. PRC2 is recruited to specific loci through interaction of SUZ12 with CTCF [82]. Another main leukemogenic interactor of PRC2 components is ASXL1. A recent study showed that ASXL1 loss affects PRC2 complexes and H3K27me3 histone marks, and induces a strong hematopoietic phenotype consistent with an MDS in a conditional knock-out mouse model [83]. ASXL1 would direct PRC2 to leukemogenic loci such as *HOXA* genes. Thus, through direct alterations of its components or of proteins or lncRNAs [84] that recruit the complex, PRC2 has emerged as a key node in a network regulating hematopoietic stem cell self-renewal and proliferation and as a major factor in myeloid leukemogenesis. This is also true for T-cell leukemogenesis [85]. Correct functioning of polycomb repressive complex 1 (PRC1) seems also to be important for myeloid cells since the loss of BMI1 (a component of PRC1) in the mouse leads to a disease similar to PMF [86]. Structural alterations of the *BMI1* gene occur but are rare in human myeloid diseases [87].

Whether other chromatin-associated complexes play a role in leukemogenesis should soon be revealed. ASXL1 could play a role in a cross-talk between major chromatin silencing systems, PRC1/PRC2, HP1α/CBX5 heterochromatin repressive complex and polycomb repressive deubiquitinase (PR-DUB) complex. Mutations in *BCOR* and *BCORL1* suggest that the RAF/BCOR complex [84,88] might be involved in AML. The recent identification of a mutation in the *DAXX* gene in an AML case [23] further supports a wide participation of chromatin-regulatory complexes in leukemogenesis and cancer in general. DAXX and ATRX (which is mutated in X-linked α-thalassemia) are subunits of a chromatin remodeling complex and are both mutated in solid tumors [89,90].

The importance of the fifth class of mutated genes was more unexpected. Mutations in components of the spliceosome, which are mutually exclusive, lead to splicing defects including exon skipping, intron retention and use of incorrect splice site [31]. A recent study showed that a consequence of splicing gene mutations is accumulation of unspliced transcripts affecting a specific subset of mRNAs [41].

#### What are the effects of the gene mutations?

The dominant-positive effects of oncogenes such as *BCR-ABL1*, mutated *FLT3, JAK2* or *RAS,* have been easy to apprehend. *CBL* and *LNK* mutations inactivate brakes on signaling pathways and may have a dominant-negative effect. *TET2* is inactivated in the manner of a tumor suppressor. *EZH2* is frequently associated with UPD and acts as a TSG. A frequent form of defect seems to be haplo-insufficiency [91], which could be associated with the (generally) heterozygous loss or mutation of *ASXL1, NF1, NPM1, TP53, RUNX1* or *TET2*. Neo-functionalization results from *IDH1/2* mutations, which are always mono-allelic. For genes altered through different mechanisms (mutations, deletions or translocations) such as *RUNX1* or with different types of mutations (hotspot or dispersed) such as *DNMT3A*, the function might be variably affected and some mutants may have a dominant-negative effect. Mutations in spliceosome genes are mostly missense and could result in proteins with a modified but not inactivated function.

Mutations in signaling pathways, transcription networks and splicing machinery have many downstream consequences. Modifications in epigenetic regulation of DNA and histones may have a strong amplifying effect since they impact on the transcription of thousands of genes. This in turn impacts on the properties of hematopoietic stem cells, favoring self-renewal and proliferation over differentiation, thus promoting leukemogenesis [92]. However, chimeric proteins involving TFs and ERs (e.g. MLL, MYST3, NSD1 …) may induce a stronger effect than mutations in other TFs and ERs (such as ASXL1, EZH2 or TET2), which may need to co-occur with several other alterations to trigger AML, often after a chronic phase. Perhaps like the difference between a water jet and a sprinkling rain, this difference may have to do with the specific functions of TFs and ERs [64]. TF and ER fusion proteins assemble in complexes that are directly recruited to their target genes where they modify the local histone marks, drastically altering transcription. In contrast, mutated ERs may moderately perturb the epigenetic network, resulting in global gene deregulation.

Mutations in spliceosome components may lead to several types of deregulation, including alterations of the epigenetic control of differentiation and self-renewal; they may thus result in the same defects as TF and ER mutations. This may derive from splicing aberrations of leukemogenic genes (e.g. *RUNX1*) [41] or from other specific but indirect defects. SF3B1 for example interacts with components of the polycomb repressor complex 1 (PRC1) and *SF3B1* mutations may compromise PRC1 regulation of leukemogenic loci [93]. Reciprocally, the function of the pre-mRNA splicing machinery involves the reading of histone marks, and defective chromatin regulators may affect splicing [94]. Directly or indirectly, *SF3B1* mutations, which are associated with the presence of ring sideroblasts, are likely to affect genes involved in red cell biology and mitochondria function. Because mutations in splicing genes, in TFs and in ERs are not mutually exclusive it is probable that the three types of alterations have additive rather than interchangeable effects.

### Modeling molecular leukemogenesis

#### Are there preferential combinations and mutual exclusions?

Two driver mutations may never occur together (mutual exclusion) in the same cell because of epistasis (two hits in the same pathway are not selected because they do not provide a growth advantage) or synthetic lethality (two hits are counter-selected because they compromise the life of the leukemic cell). Associations and cooperation can occur in all other cases.

Some chronic myeloid malignancies, such as CMML (myeloproliferative form, MP-CMML) and MPNs, have a proliferative component. This component is driven by alterations in signaling molecules, such as *CBL, CBLB, FLT3, JAK2, LNK, MPL, NF1, PTPN11* or *RAS*. These mutations are generally mutually exclusive. However, *JAK2* mutations can be found in patients with mutations of *CBLLNK* or *MPL*[95-97]. In most cases when two signaling mutations are found in the same patient they are not in the same cellular clone. Signaling mutations associate with mutations in genes from the other classes (TSGs, TFs, ERs). *CBL* and *KIT* mutations are more frequent in AML with t(8;21) and inv(16), i.e. with alterations of the core binding factor (CBF), a dimeric transcriptional factor containing the RUNX1 protein [98].

With rare exceptions, mutations in genes encoding splicing factors do not synergize and are mutually exclusive [31,38,41,42].

As already mentioned, *IDH1* or *IDH2* mutations are mutually exclusive with *TET2* mutations. Except for this, *TET2* mutations seem to be able to cooperate with either of the other recurrent alterations. *ASXL1* mutations, which occur preferentially in secondary AML, are mutually exclusive with *NPM1* mutations, which occur in *de novo* AML [99]. Although ASXL1 interacts with PRC2 proteins [83]*ASXL1* and *EZH2* mutations are not mutually exclusive [58]. Mutations in *EED* and *SUZ12* may even be found in the same AML case [23]; however, they may affect different clones. *RUNX1* mutations are frequently associated with *ASXL1* defects in MDSs [100]. Mutations in *ASXL1* and *TET2* can be concomitant (Figure 1), and each can co-occur with mutations in signaling molecules [58,100]. In MDSs, *U2AF1* mutations are more frequent in *ASXL1*-mutated than in *ASXL1*-wildtype cases [38,42]. *TP53* mutations and losses, likely associated with genetic instability, are found in MDSs with karyotypic alterations but not in cases with normal karyotype [58]. *DNMT3A* mutations are more frequent in AML with *NPM1* and *FLT3* mutations, infrequently found in *ASXL1*-mutated cases, and very rare in cases with translocations [24,101]. Overall, while *IDH1/2* and *TET2* mutations are equally distributed, there seem to be two major associations in AMLs with intermediate cytogenetic risk, *ASXL1/RUNX1* on the one hand (secondary, dysplastic AMLs), *NPM1/FLT3/DNMT3A* on the other hand (primary, non-dysplastic AMLs) [99]. These and other associations and exclusions not described here or yet to be discovered will help understand the major leukemogenic pathways. An important issue is to demonstrate that mutations found in the same case are actually cooperating mutations that co-occur in the same cell progeny and not in different clones.

#### How many hits are necessary to trigger a malignant myeloid disease?

Early studies of chronic and acute hematopoietic malignant diseases have shown that some cases may display a single mutational event whereas others harbor several hits [100]. This difference may just be due to the low mutational frequency of many driver genes (e.g. *NF1A, EED*) [102] and to our current ignorance of other targets. Actually, NGS studies have shown that the general rule is to find several altered genes in each case [23,26,59,103] and murine models have shown that single alterations are, except in rare cases, not sufficient to cause AML [104,105]. In the years to come mouse models will have to challenge many combinations of mutations.

The study of matched chronic and acute stages has shown that progression is associated with additional alterations. However, the chronic stages are already characterized by the presence of several mutations. We found that many cases of CMML have already four mutations [36], and this was without counting mutations in splicing factors. *JAK2* and *TET2* concomitant mutations are frequent in MPNs [16,37]. Whether they are both necessary for the various phases of the disease and their order of appearance are a matter of debate [106]. An NGS study indeed showed that the ten mutations identified in an MDS patient can be detected together in most studied single cells, suggesting a linear evolution of the disease and the existence of a dominant clone [103]. Regarding evolution of AML after therapy, a recent NGS study has revealed two major patterns at relapse [23]; the first pattern is the persistence of a dominant clone and the second pattern is the selection and expansion of a minor clone; in both cases the relapse clone had gained additional mutations. Another recent NGS study showed that genetic evolution of secondary AML is a dynamic process shaped by multiple cycles of mutation acquisition and clonal selection. MDS are oligoclonal with founding clones; these clones persist in secondary AML, which shows at least an additional subclone with new “progression” mutations [107]. Founding mutations may occur in various genes, such as *U2AF1*[39] or *TET2*. Many different genes may be involved in progression. Thus, several steps are necessary to trigger a myeloid disease, even a so-called chronic one, and progression involves additional hits.

#### How many of these steps are there?

A first step in the leukemogenic process is likely to be a mere clonal expansion. Several gene mutations may play a role at this stage. Their identity may depend on whether they target a hematopoietic stem cell or a progenitor. In the first case the initial hit should provide a proliferation boost, in the second the hit should bestow self-renewal on the proliferating progenitor [108]. Mutations in a TSG, splicing gene, or in some ERs such as *TET2,* could occur at this initial step. It is also possible that, in a susceptible background, several clones emerge independently early on [12,109].

Then, because of increasing proliferation and genetic instability, a cell from the affected clone (or clones) undergoes various additional mutations (including many background mutations), leading to an oligoclonal malignant tumor. Some of the early mutations may not be present in the clone that eventually becomes leukemic. Thus, for each case, only the determination of all potential mutations and the reconstitution of the mutation profile and clonal evolution will help understand the pathophysiology of the disease. This is now achievable by using NGS. How many steps can eventually be individualized may depend on how many clones are initially expanded, on the level of genetic instability that results from the initial hits, and on the impact of the mutations on self-renewal, differentiation and proliferation. Some mutations in epigenetic regulators may have a milder effect on genetic reprogramming than a gene fusion involving a master transcription factor, which will induce a strong block of differentiation in a hematopoietic precursor [92]. The latter event is prominent in *de novo* AMLs, which accordingly display only few or none of the other recently-discovered mutated genes.

A previous scheme of leukemogenesis [110] was based on the minimal cooperation of two oncogene classes, proliferation-drivers (kinases, RAS) and differentiation-blockers (mostly transcription factors), to trigger AML. The ever-increasing molecular complexity of myeloid malignancies is now obvious and calls for an update of this model. First, it is now routinely possible to observe the cooperation, already at the chronic stage, of three, four or more mutated genes (to speak only of known or suspected drivers), whose products belong to at least five classes, class I signaling molecules class II TFs, class III ERs, class IV TSGs and class V splicing factors [100]. Second, not all mutations of a class are equivalent; mutations in *ASXL1, RUNX1* or *TET2* occur almost as frequently at the chronic stages as in AML whereas mutations of *IDH1/2* or *DNMT3A* are preferentially found at the acute stage. The reason for this remains obscure but may have to do with the different intensities in the differentiation block induced by the mutations. Third, the classes are not well individualized. For example, *EZH2, RUNX1* and *TET2* are both TSGs and TF/ERs. *NF1* is both a TSG and a regulator of signaling pathways. Because it induces phosphorylation of histone H3 and PRMT5 arginine methyltransferase, JAK2V617F may also be an ER [111]. Fourth, all classes may not be systematically affected in each case. Fifth, if classes I and V are relatively well individualized, with genes whose mutations are generally mutually exclusive, the definition of the other classes may evolve. However, despite all this, the initial schematic model might not be so far off. The two key processes of differentiation and self-renewal seem to be always altered and proliferation is frequently affected. It may just be that the oncogenic hits required to achieve each step might be more numerous than initially expected. This model will apply to cases with intermediate or normal cytogenetic risk; a different leukemogenesis pathway linked to genetic instability may be involved in cases with *TP53* mutation and complex karyotype [112].

**Figure 4:**
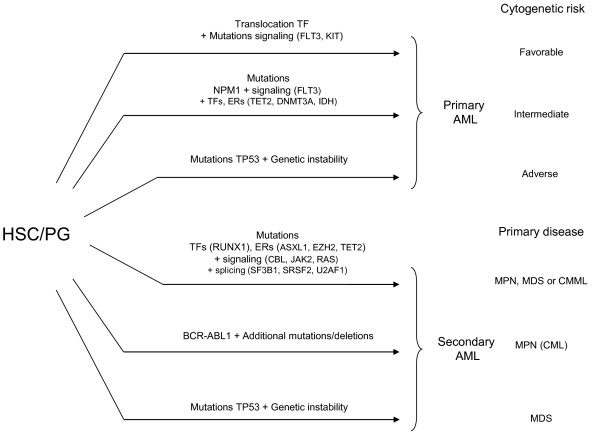
**Schematic representation of pathways leading to acute myeloid leukemia (AML) from hematopoietic stem cell (HSC) or progenitors (PG).** Gene fusions and *NPM1* mutation are major events in the induction of primary AMLs with favorable and intermediate cytogenetic risk (they correspond respectively to mutation groups A and B of Ley et al [24], and to mutation groups 2 + 3 and 1 of Shen et al. [101]. Secondary AML following MPN or MDS (see Figure 1) could occur after a series of gene mutations in transcription factors and epigenetic regulators combined with a mutation in a signaling pathway (see Figure 3), after TP53 mutation and a series of mutations and karyotype alterations due to genetic instability, or after additional mutations in BCR-ABL chronic myeloid leukemia.

**Figure 5:**
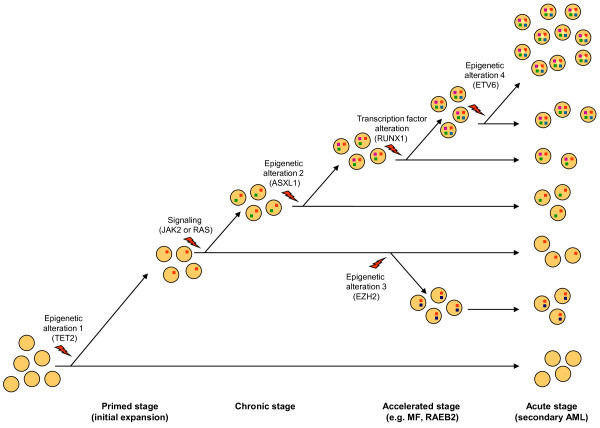
**Schematic representation of a case of malignant myeloid disease evolving in four stages along one pathway.** Clones with different gene mutations (color squares in cells) represent various ratios of the oligoclonal leukemia. The order and nature of the mutations (or genome alterations) is given as an example and may differ from one case to another. However, in contrast to JAK2V617F, which has a mild effect on hematopoietic stem cell (HSC) [16], *TET2* mutation has the property to initiate the amplification of HSC and to pave the way to secondary mutations [77]. Mutations in signaling molecules, which have a major impact on the disease phenotype, will vary with the type of chronic stage, for example it could affect *JAK2* in case of MPN, *RAS* in case of MP-CMML and be absent in case of MDS. MF: myelofibrosis, RAEB: refractory anemia with excess of blasts, AML: acute myeloid leukemia.

Considering all this, several pathways to leukemia can be envisaged (Figure 4). The first pathway could be direct and trigger *de novo* AML with a gene fusion as the major event and few other alterations. The second pathway is characterized by *NPM1* mutations, which are rarely associated with mutations in other known TFs or ERs except in *DNMT3A* and *IDH1/2*[23,101]. AML with complex karyotype can derive from genetic instability, with or without *TP53* mutations. A fourth pathway would be the accumulation of several hits in signaling molecules, TFs, ERs and splicing factors, which induce either secondary AML after a chronic phase (Figure 5) or *de novo* AML; however, some so-called *de novo* AMLs with several ER mutations could actually be secondary to a non-detected chronic phase. Mutations in TFs and ERs are not major events in chronic myeloid leukemia (CML), which is triggered by the *BCR-ABL1* fusion; however, mutations in ERs such as *ASXL1, IDH1/2* and *TET2* may participate to CML progression to AML [113,114].

**Figure 6:**
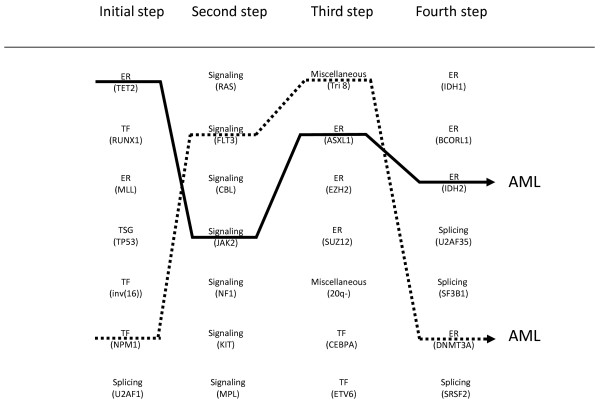
**“Slot machine” model of leukemogenesis.** Alterations in signaling molecules, transcription factors (TFs), epigenetic regulators (ERs), tumor suppressors (TSG), spliceosome components and various genome abnormalities (examples are given) fall into (at least) four “reels” (steps) that combine to induce a malignant myeloid disease. Acute myeloid leukemia (AML) results from one of the allowed combinations of four (at least) cooperating alterations. At chronic stages, the steps are variably combined, some may be absent (e.g. signaling), some may be specific (e.g. *SF3B1* splicing mutations in RARS). Each step can be achieved by alterations in one of several genes. The initial step leads to expansion of a founding clone. Two examples of draw (plain and dotted lines) leading to AML are shown.

Overall, the development of an AML may follow a “slot machine” model (Figure 6), in which the late steps would be, to some point, constrained by the initial ones (clonal dominance, cooperations/exclusions). Oligoclonality would be due to several possible draws at each step. It is important that we determine the exact number of “reels” (hits) and “symbols” (genes) and the possible combinations.

### Utilizing molecular leukemogenesis

Understanding and modeling leukemogenesis will have a major impact on the management and treatment of hematopoietic malignancies. Molecular biology already helps establish the diagnosis (*JAK2*), classification (*BCR-ABL1, FGFR1, PDGFRs*), prognosis (*FLT3, NPM1, CEBPA*) and treatment (BCR-ABL, 5q-, JAK2) of myeloid diseases. Due to the increasing simplification and accessibility to clinical laboratories of NGS equipment, the repertoire of all genetic alterations will soon be determined for any new case as a routine practice.

The establishment of a precise taxonomy comprising homogeneous pathophysiological entities is a major goal in hematology. It relies heavily on molecular data. It started with the karyotype and has continued with gene expression profiles [115,116]. Gene mutations will nicely complete the picture. Other factors such as microRNAs and long non-coding RNAs status [1], methylation profiles [117] and histone marks may have to be integrated too.

Several studies have shown that gene mutations have indeed a major impact on prognosis of myeloid diseases. This is the case in MDSs for mutations in five genes, *ASXL1, ETV6, EZH2, TP53* and *RUNX1*[58]. Mutations in *ASXL1* seem to be associated with an aggressive phenotype in all myeloid malignancies [8]: they are frequent in high-risk MDSs and correlate with poor prognosis in MDSs [118-120] and with acute progression in CMML [36], they are more frequent in myelofibrosis than in other MPNs [37,121], and characterize secondary AML. *DNMT3A* mutations are frequent in younger patients with AML and are associated with an unfavorable prognosis in MDS and AML [15,24,30,101]. Among splicing gene mutations, those in *U2AF1* and *SRSF2* seem to be associated with aggressive forms of myeloid diseases and those in *SF3B1* with good prognosis [38,39,41,42]. Molecular data will allow the establishment of an upgraded index of prognosis. For example, in MDSs, it is highly conceivable that the current prognostic index used for the evaluation of the disease (IPSS), which already includes karyotypic data, can be improved by a molecular index regrouping the mutations that impact on the patient’s outcome [112]. Whether *TET2* mutations are to be included is a matter of debate [122-124]. In AML, a thorough study of 18 genes, including *ASXL1* and *DNMT3A*, proposed an updated and precise risk stratification based on gene mutations [125].

New therapeutic targets can be found in two of the five major classes of leukemogenic genes. Following the successful use of imatinib in CML, abnormal signaling pathways associated with myeloproliferation, be it the JAK-STAT pathway [126-128] or another pathway, represent appealing targets. Drugs targeting epigenetic modifications, i.e. epidrugs, such as histone deacetylase inhibitors and hypomethylating agents (DNMT inhibitors), are currently developed or used in clinics, and many new ones are studied in preclinical assays and clinical trials [1]. Targeting histone methyltransferases (e.g. MLL) or lysine acetyltransferases (e.g. P300) [129] is also a promising area of development. The determination of gene mutations and their consequence on gene regulation and cell programming will help treat myeloid malignancies in providing a rationale for the use and development of new epidrugs, in directing the choice of the drug cocktails, and in allowing the design of drug delivery and the monitoring of drug response and disease progression. For example, agents directed against TET2-, IDH- and DNMT3A-associated methylation defects may represent a new area of development. To date, the use of *TET2* mutations status to evaluate the response to DNMT inhibitors is still debated [130,131]. Because many mutations compromise PRC2 function drugs antagonizing this defect hold great promise.

Proteins of two other leukemogenic classes may also serve as therapeutic targets. For example, the antitumor macrolide pladienolide targets SF3B1 [132] opening new opportunities to develop treatments against RARS. Compounds aiming at restoring a normal P53 pathway are in development [133,134].

The existence of concomitant mutations is an incentive for combinatorial therapies; for example, therapeutic synergy may be obtained by the combined use of signaling inhibitors and epidrugs.

Finally, the complete determination of the mutation repertoire will provide novel therapeutic targets. For some diseases, such as CMML, it is already possible to identify at least one target for nearly nine cases out of ten [33,36]. However, the development of resistance, as observed with imatinib [135], is a critical issue. Hopefully, target identification will allow for the development of new combinatorial strategies, such as the one based on synthetic lethality [136,137]. If two mutations never occur together it may mean that their combined effect is deleterious. Thus, opportunities for deriving synthetic lethality drugs could stem from the observation of exclusions in mutations patterns.

## Conclusions

Thus, mutations and models (“M and Ms”) will help manage myeloid malignancies. The eventual comprehensive determination in any given case and at diagnosis, of the set of altered genes, underlying affected pathways and disease stage, will guide towards an optimal treatment based on an appropriate combination of drugs targeting the various affected processes of the disease. Clinically-oriented laboratories should already be preparing for that challenge. Meanwhile, there is much to mull over the “M and Ms” of myeloid malignancies.

## Competing interests

The authors have no competing interests.

## Author’s contributions

All authors have contributed ideas, discussions, and have participated in the writing of the manuscript. All authors read and approved the final manuscript.

## Pre-publication history

The pre-publication history for this paper can be accessed here:

http://www.biomedcentral.com/1471-2407/12/304/prepub
